# *De novo* transcriptome assembly of a lipoxygenase knock-down strain in the diatom *Pseudo-nitzschia arenysensis*

**DOI:** 10.1038/s41597-024-03375-0

**Published:** 2024-05-22

**Authors:** Pina Marotta, Valeria Sabatino, Luca Ambrosino, Marco Miralto, Maria Immacolata Ferrante

**Affiliations:** 1 Integrative Marine Ecology, Stazione Zoologica Anton Dohrn, Villa Comunale, 80121 Naples, Italy; 2 Department of Research Infrastructures for marine biological resources, Stazione Zoologica Anton Dohrn, Villa Comunale, 80121 Naples, Italy; 3 Associate to the National Institute of Oceanography and Applied Geophysics, 34151 Trieste, Italy; 4 Present Address: Institute of Biochemistry and Cell Biology, National Research Council of Italy, Via P. Castellino 111, Naples, 80131 Italy

**Keywords:** Genetics, Computational biology and bioinformatics

## Abstract

Diatoms are microalgae that live in marine and freshwater environments and are responsible for about 20% of the world’s carbon fixation. Population dynamics of these cells is finely regulated by intricate signal transduction systems, in which oxylipins are thought to play a relevant role. These are oxygenated fatty acids whose biosynthesis is initiated by a lipoxygenase enzyme (LOX) and are widely distributed in all phyla, including diatoms. Here, we present a *de novo* transcriptome obtained from the RNA-seq performed in the diatom species *Pseudo-nitzschia arenysensis*, using both a wild-type and a LOX-silenced strain, which will represent a reliable reference for comparative analyses within the *Pseudo-nitzschia* genus and at a broader taxonomic scale. Moreover, the RNA-seq data can be interrogated to go deeper into the oxylipins metabolic pathways.

## Background & Summary

Diatoms are a group of very diverse photosynthetic microorganisms that evolved a broad range of adaptive strategies allowing them to prosper under a wide variety of temperature, light, and nutrient conditions^1^. Thanks to these characteristics, they populate almost all aquatic and wet environments, contributing to ca. 20% of global carbon fixation^1,2^; additionally, as important primary producers, they form the basis of aquatic food webs. The genus *Pseudo-nitzschia* is one of the most common genera of diatoms, comprising about 60 worldwide distributed species, among which 26 species produce the neurotoxin domoic acid and are responsible for harmful algal blooms^3^. Among the species of this group, *P. arenysensis*^4^ is a non-toxic species that regularly blooms in coastal and oceanic waters^5^; its life cycle has been well described^6^ and the transcriptome and an optimized transformation protocol are already available^7–9^. All these characteristics made this species a good model for functional and comparative genomic studies.

The high rate of biodiversity characterizing diatoms makes them producers of high-value bioactive compounds, whose identification could be exploitable by the biotechnology industry^10,11^, such as oxylipins, oxygenated fatty acids involved in the reduction of grazing pressure^12,13^, in the chemical communications regulating phytoplankton dynamics^14–16^ and the interactions with bacteria^17^. Lipoxygenase enzymes (LOXs), a group of nonheme iron-containing dioxygenases, are responsible for the biosynthesis of these metabolites^18–23^. Distinct oxylipins are produced by different LOXs, depending on the polyunsaturated fatty acid (PUFA) used as substrate and the position on the carbon backbone where oxygen (O_2_) is added^18^.

*Pseudo-nitzschia* members produce a wide range of species-specific oxylipins^24^, suggesting differentiation of LOX enzymes to ensure to these metabolites a species-specific mediator role in the plankton community^24,25^. To investigate the ecological roles of these secondary metabolites, studies that examine their structure and biosynthesis should be paralleled with functional studies on the enzyme-coding genes involved in their synthesis. In recent work, we explored the role of LOX in *P. arenysensis* (*PaLOX*)^26^, a diatom known to synthesize oxylipins throughout both 12- and 15S-LOX pathways^14,16,24^. Taking advantage of sequence information from the Marine Microbial Eukaryote Transcriptome Sequencing Project (MMETSP)^7^ we discovered that a unique LOX transcript is present in this species and generated mutants in which this gene was silenced^26^.

Here, we describe the *de novo* transcriptome assembly of a LOX-interfered *P. arenysensis* clone and of the corresponding wild-type strain, which captures two different conditions with respect to that pictured by the *P. arenysensis* transcriptome sequenced within the MMETSP^7^. The availability of this new transcriptome, built by exploiting new datasets and upgraded pipelines and software, is useful because it allows to enrich the information of previously assembled transcriptomes by adding or updating missing or badly annotated transcripts^27^. Furthermore, to elucidate the molecular mechanisms and the gene networks underlying diatoms adaptability, the “-omics” technologies have spread considerably, and the genome of an ever-increasing number of diatom species, among which *P. arenysensis*, is being sequenced. Within this context, the 12 RNA-seq datasets released with this paper, together with previously generated datasets for the same species, will aid in building accurate gene models and will allow to enrich the species gene expression atlas. In addition, the integration of the information deriving from the genome sequence analysis, the transcriptome of different strains, the gene expression profiles, and the comparison with similar data from the other diatom species will shed light on key genes mediating adaptation across the global ocean. Since the timing for the genome release is still unknown, the present transcriptome represents a resource allowing more accurate comparative genomic analyses within the *Pseudo-nitzschia* genus and at a broader taxonomic scale.

Moreover, we already demonstrated that the oxylipin reduction in *P. arenysensis* results in growth impairment of the interfered cells compared to the WT^26^, and the RNA-seq data can be interrogated to reveal the underlying perturbated pathways. Finally, the identification of *P. arenysensis* molecular mechanisms tuning the oxylipin-mediated cell growth regulation can be used as a guide in determining a correlation between diatom cell growth/density and oxylipin concentrations in other diatom species.

## Methods

### Culture growth condition and RNA preparation

**Fig. 1:**
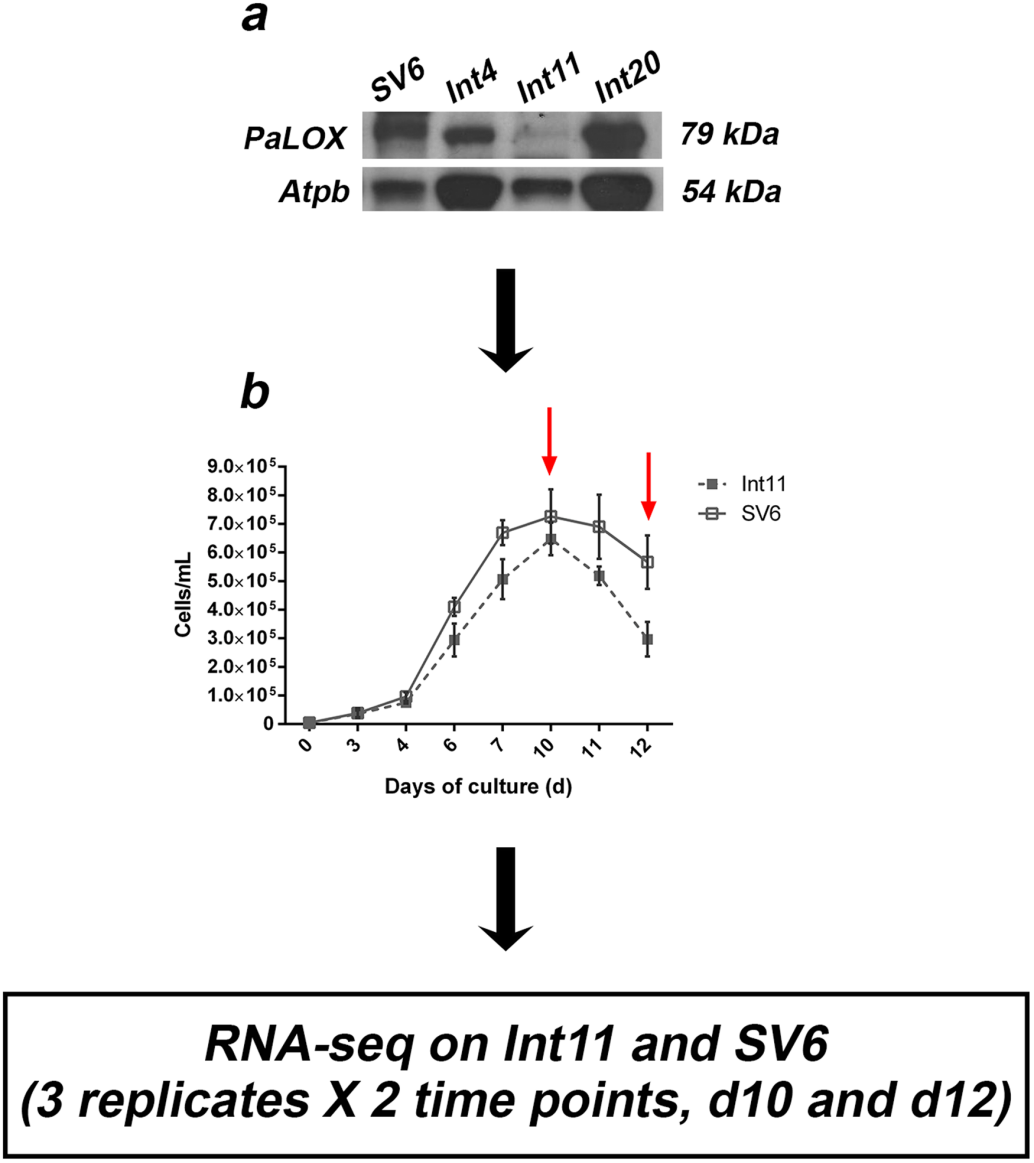
Diagram illustrating the experimental design. (**a**) Western blot analysis of the *P. arenysensis* LOX protein, PaLOX, performed on the wild-type (SV6) and three silenced samples (Int4, Int11, Int20); the beta subunit of the ATP synthase, Atpb, was used as an internal control. LOX antibodies bind to a protein of c. 79 kDa, AtpB antibodies bind to a protein of c. 54 kDa; (**b**) Growth curve of the transformant Int11 (dotted line/closed square) with its control SV6 (solid line/open square). Red arrows indicate sampling points for RNA-seq, day 10 (T10) and day 12 (T12). At those time points, the growth of the transformant strain compared to the control was reduced of approximately 11% and 50%, respectively. Mean values obtained from three biological replicates and SD are presented.

The wild-type SV6 strain of *P. arenysensis* was obtained from crosses performed in the laboratory from strains isolated at the Mare Chiara LTER station in the Gulf of Naples. The LOX-interfered *P. arenysensis* Int11 sample, derived from the biolistic transformation of SV6 cells, has been described in Sabatino *et al*.^26^. Cultures were grown in seawater enriched with F/2 nutrient^28^ and incubated at 18 °C under white light at approximately 70 μmol photons m^−2^ s^−1^ and 12:12 h dark:light cycle. For the growth curves, fresh diatom cultures were inoculated at a start cell density of 5000 cells mL^−1^, grown under the above-mentioned conditions. The growth was monitored daily by cell counting under a Zeiss inverted microscope and using Malassez chambers of 100 μL capacity. Each curve was performed in triplicate. Cells were collected at two time points of the growth curve, the stationary phase (T10) and the senescence phase (T12) (Fig. 1 and Supplementary Fig. 1). The RNA extraction was performed following the protocol detailed in Amato *et al*.^29^. Specifically, mutated and wild-type *P. arenysensis* cultures were filtered onto RAWP Millipore cellulose membrane filters (Mf-Millipore RAWP04700, 1.2 µm porosity) and washed with MilliQ water. Filters were put into 2 ml Eppendorf tubes filled with Roche TriPure® isolation reagent (Merck KGaA, Darmstadt, Germany), snap-frozen in liquid nitrogen, and stored at −80 °C until use. TriPure®-soaked filters were thawed at room temperature and RNA extraction was conducted according to the manufacturer’s instructions. RNA samples were DNAse-treated (TURBO DNA-free™ Kit, Waltham, Massachusetts, USA) to get rid of genomic DNA contamination and purified with Qiagen RNeasy Mini Kit (Qiagen, Hilden, Germany). PCRs were performed using the RNA as template to verify absence of genomic DNA contamination. Samples were stored at −80 °C until use.

### Quality control of total RNA samples, library preparation and sequencing

RNA samples were analyzed on an Agilent 2100 Bioanalyzer platform (Agilent Technologies 5301 Stevens Creek Blvd. Santa Clara, California 95051 USA) to assess integrity, on a NanoDrop 2000 Spectrophotometer (Thermo Fisher Scientific Inc., Waltham, Massachusetts, USA) to assess purity, and quantified with a Qubit fluorometer (Thermo Fisher Scientific Inc.). 300 ng of RNA from each sample per time point was used to produce libraries with the Illumina TruSeq original protocol, with a bead-based poly-A capture approach, and sequenced on an Illumina HiSeq 2000 (Single End 50 bp reads) at the GeneCore facility of the European Molecular Biology Laboratory (EMBL).

### Reads quality check, transcriptome assembly and gene annotation

The quality check of the raw reads was performed using FASTQC^30^. A trimming step was carried out by Trimmomatic^31^, setting a minimum lentgh of the reads to 30 bp and using the “ILLUMINACLIP” parameter to remove TruSeq Adapters. All the cleaned reads were assembled into transcript sequences using Trinity v.2.15^32^ with *in silico* read normalization, setting the -min_kmer_cov parameter at 2. The clustering of the transcriptome was performed using the CD-hit software v. 4.6.8^33^ with 90% identity threshold in order to remove transcriptome redundancy. Since we obtained a partial assembly of the lipoxygenase (LOX) transcript, the complete assembly of the LOX was performed by Spades v.3.15.4^34^, setting the k-mer size to 23 and using the LOX sequence obtained by previous experiments^26^ as the object of the “–trusted-contigs” parameter. 695 sequences from bacteria or viruses, identified via the Transcriptome Shotgun Assembly (TSA) web portal at NCBI^35^, were filtered out. Moreover, one transcript sequence with less than 200 bases length was also removed. The completeness of the transcriptome was evaluated by using Busco v.5.7.0^36^, setting *stramenopiles* as the lineage of search. The whole transcriptome was aligned with BLASTx software^37^ versus the Uniprot/SwissProt database^38^ (downloaded in September 2022), setting the e-value threshold to 1e^−3^, and retrieving the best hit for each assembled transcript.

### Expression analysis

Cleaned reads were mapped on the assembled transcriptome with bowtie v.2.3.4.1^39^. Reads counts for each replicate were performed using the eXpress software^40^. The hierarchical clustering dendrogram was obtained with the WGCNA package in R^41^, setting an “average” distance parameter in the *hclust* function. The principal component analysis (PCoA) was performed by Scikit-learn module^42^ in Python programming language^43^, and plotted by using *seaborn*^44^.

## Data Records

All the RNA-Seq raw reads generated in this project were deposited in the NCBI Sequence Read Archive database with identifier SRP408880^45^, under project identification number PRJNA903772. The *de novo* transcriptome assembly resource (final transcriptome) is available in the NCBI Transcriptome Shotgun Assembly with accession GKNO00000000^46^ and in the Zenodo entry, where we also added the annotated genes file as XLS file^47^.

## Technical Validation

### Quality control

RNA-seq experiments^45^ were performed on two strains of *P. arenysensis*; in detail, six samples of a wild-type strain culture and six samples of an interfered strain culture were collected (two time points in triplicate per strain). The pre-trimming FASTQC step enabled us to identify the TruSeq adapters to be removed in the next cleaning step. The trimming procedure removed all the reads shorter than 30 bp, together with the identified TruSeq adapters. The post-trimming FASTQC step allowed us to check that all the trimmed reads retained a minimum quality PHRED score of 30.

### Transcriptome assembly and annotation

Trimmed reads have been assembled into a transcriptome^46^ with a *de novo* approach. The assembled transcriptome accounted for a total of 31758 transcripts (Table 1), with a mean GC content of 46.01%, an average and median contig length of 925.58 and 681 bp, respectively, and a N50 of 1417 bp (Table 1). The assembled transcriptome was subjected to a clustering procedure in order to remove redundancy. Moreover, a step to filter out sequences from bacteria or viruses and sequences shorter than 200 bases in length was performed, removing 696 sequences. After this step, the final transcriptome accounted for a total of 27784 transcripts, in the same range of the MMETSP *P. arenysensis* transcriptome^7^ (MMETSP0329). The procedure of functional annotation enabled the functional classification of 8857 transcript sequences (31.9% of the total transcriptome, Table 1).

**Table 1:** Basic statistics of the assembled transcriptome of *P. arenysensis*.

Number of Trinity transcripts	31758
Number of Trinity genes	29337
Trinity assembly GC %	46.01
Trinity assembly mean length	925.58
Trinity assembly median length	681
Trinity assembly N50	1417
Total Trinity assembled bases	29394633
Number of transcripts after clustering	28480
Number of filtered out transcripts	696
Number of final transcripts	27784
Functional annotated transcripts	8857

A summary of the number of reads obtained from the sequencing step and their mapping on the assembled transcriptome is shown in Table 2.

**Table 2:** Reads and mapping information for the *P. arenysensis* RNA-seq samples.

File	Raw reads	Cleaned reads	Mapped reads %
WT1-T10	18167102	17907604	96.57
WT1-T12	19298580	19229237	94.72
WT2-T10	20172929	19974744	97.17
WT2-T12	22603520	22417183	96.45
WT3-T10	18129088	18076439	96.89
WT3-T12	19970146	19854771	95.82
INT1-T10	24164247	24033975	96.48
INT1-T12	18653532	18592969	93.37
INT2-T10	23258748	23166059	96.30
INT2-T12	20788118	20752811	94.17
INT3-T10	25590601	25472967	96.79
INT3-T12	18624590	18453390	93.87

**Fig. 2:**
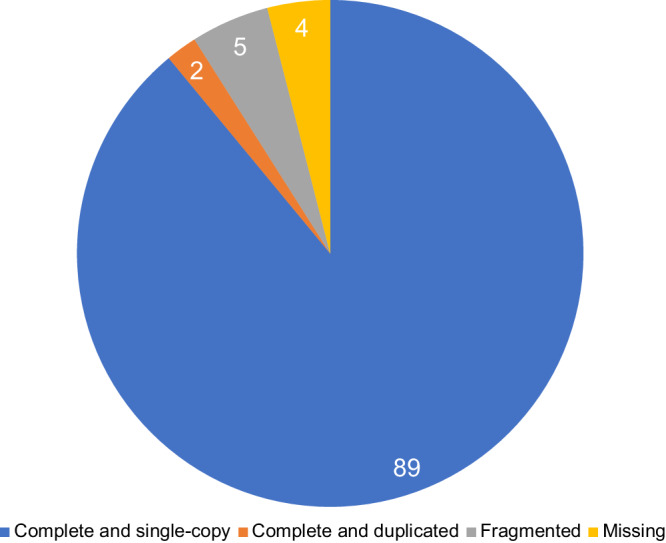
Summary of detected *stramenopiles* BUSCO genes. The number of complete (single-copy and duplicated), fragmented and missing genes are reported.

The BUSCO analysis revealed that the transcriptome has 91 complete *stramenopiles* BUSCO genes over 100 total genes (89 single-copy and 2 duplicated genes), with only 4% completely missing (Fig. 2).

### Preliminary expression results

**Fig. 3:**
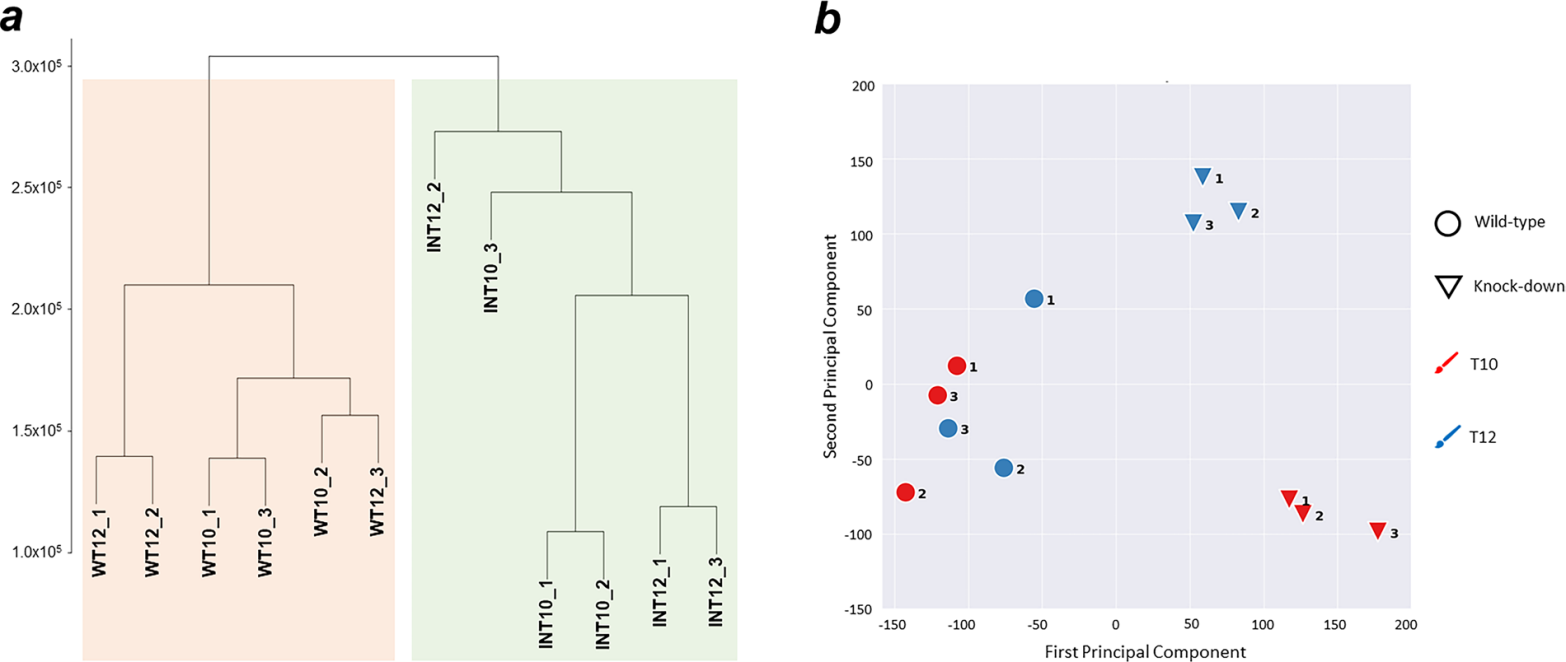
RNA-seq samples similarity analysis. (**a**) Dendrogram of hierarchical sample clustering, wild-type samples are highlighted by the pink box, and knock-down samples are highlighted by the green box. (**b**) Principal component analysis of RNA-seq samples; different sample categories are indicated according to the legend.

The sample clustering of the expression levels of each replicate revealed a sharp distinction between wild-type and knock-down samples (Fig. 3a). The principal component analysis revealed a similar clear discrimination between wild-type and interfered samples, together with a well-defined separation within the knock-down samples between the stationary phase (T10) replicates and the senescence phase (T12) replicates (Fig. 3b).

## Usage Notes

The transcriptome^46^ presented here represents an alternative to the MMETSP^7^ *P. arenysensis* transcriptome, and together with it, it allows to extract information from two different strains from the same geographical location (B593 and SV6) and different physiological conditions (SV6 wild-type and SV6 PaLOX-silenced). Compared to BUSCO statistics of the MMETSP transcriptome (60 complete single-copy genes, 10 missing genes), the transcriptome we presented improved the number of complete single-copy genes (89) and reduced the number of missing genes (4). A summary of the transcriptome statistics is shown in Supplementary Table 1 (S1). All these resources will be useful for a high-quality gene model prediction in the perspective of sequencing the *P. arenysensis* genome^48^, which in turn will be extremely useful for comparative genomics studies when other diatom genomes are released, such as those planned within the “100 Diatoms Genomes Project” at the Joint Genome Institute (JGI)^49^. Moreover, our data could be an important reference in large-scale metagenomic and metatranscriptomic data analyses of eukaryotic plankton in the open ocean^5^ and coastal ecosystems, such as those collected within the TARA Oceans^50^ and TREC (https://www.embl.org/about/info/trec/) expeditions, respectively, or the augmented observatory NEREA (https://www.nerea-observatory.org/). Finally, transcriptome data from the LOX-interfered *P. arenysensis* strain^45^ provide a foundation for future detailed studies on the oxylipin-mediated cell signaling pathways in this and in other diatom species, while the availability of different *P. arenysensis* RNA-seq datasets could also be useful to uncover single nucleotide polymorphisms (SNPs) in the coding regions of the genome.

### Supplementary information

Supplementary Figure and Table

## Data Availability

The article includes a list of software programs, such as de novo transcriptome assembly, pre- and post-assembly procedures, and transcriptome annotation, all of which are specified alongside their respective versions within the Methods section. If specific parameter details are not provided, the programs were used with their default settings.
