# Supplementary material for: De novo transcriptome assembly of a lipoxygenase knock-down strain in the diatom Pseudo-nitzschia arenysensis
Source: Sci Data. 2024 May 22;11:522. doi: 10.1038/s41597-024-03375-0 (PMC11111692; doi:10.1038/s41597-024-03375-0)
Supplement: Supplementary file 1 — Supplementary Figure and Table [file 41597_2024_3375_MOESM1_ESM.pdf]

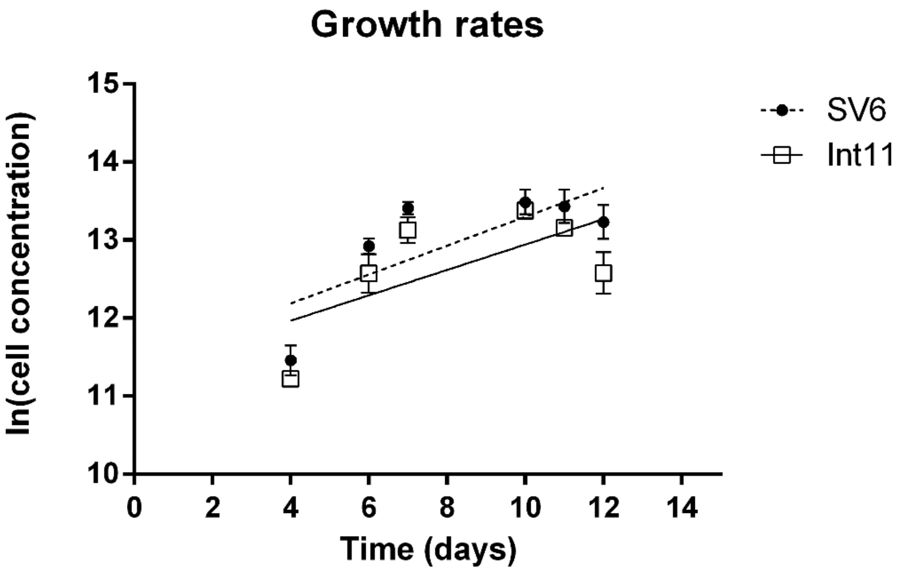

Supplementary Figure 1. Natural log of cell concentration over time for SV6 and Int11 cultures. A linear regression is fitted across the exponential phase, with the slope giving the growth rate of the cultures (SV6: 0,1849 ± 0,04286; Int11: 0,1626 ± 0,04864).

	New assembly	*MMETSP0329*
Number of transcripts	27784	21373
GC %	45.94	47.84
Mean length	962.04	1463.75
Median length	742	1218
N50	1435	1878
Total assembled bases	26729384	31284825
Complete single-copy BUSCO genes %	89	60
Complete duplicated BUSCO genes %	2	25
Fragmented BUSCO genes %	5	5
Missing BUSCO genes %	4	10

Table S1. Transcriptome statistics of the newly assembled *P. arenysensis* final transcriptome (after clustering and filtering) and of the MMETSP assembly (statistics estimated on file MMETSP0329.cds.fa, available at http://www.imicrobe.us/#/samples/1815).
